# *Entamoeba histolytica*—Gut Microbiota Interaction: More Than Meets the Eye

**DOI:** 10.3390/microorganisms9030581

**Published:** 2021-03-12

**Authors:** Serge Ankri

**Affiliations:** Department of Molecular Microbiology, Ruth and Bruce Rappaport Faculty of Medicine, Haifa 31096, Israel; sankri@technion.ac.il

**Keywords:** gut microbiota, *entamoeba histolytica*, resistance to oxidative stress, resistance to nitrosative stress, virulence

## Abstract

Amebiasis is a disease caused by the unicellular parasite *Entamoeba histolytica*. In most cases, the infection is asymptomatic but when symptomatic, the infection can cause dysentery and invasive extraintestinal complications. In the gut, *E. histolytica* feeds on bacteria. Increasing evidences support the role of the gut microbiota in the development of the disease. In this review we will discuss the consequences of *E. histolytica* infection on the gut microbiota. We will also discuss new evidences about the role of gut microbiota in regulating the resistance of the parasite to oxidative stress and its virulence.

## 1. Introduction

Amebiasis is caused by the protozoan parasite *Entamoeba histolytica*. This disease is a significant hazard in underdeveloped countries with reduced socioeconomic and poor sanitation. It is assessed that amebiasis accounted for 55,500 deaths and 2.237 million disability-adjusted life years (the sum of years of life lost and years lived with disability) in 2010 [1]. Amebiasis has also been diagnosed in tourists from developed countries who return from vacation in endemic regions. Inflammation of the large intestine and liver abscess represent the main clinical manifestations of amebiasis. Amebiasis is caused by the ingestion of food contaminated with cysts, the infective form of the parasite. Following excystation, the trophozoites migrates to the large intestine resulting in either asymptomatic colonization (90% of all infections) or causing bloody diarrhea. For unknown reasons, the trophozoites can become virulent and invasive, cause amebic dysentery, and migrate to the liver via the portal veins, where they cause hepatocellular damage. No vaccine against amebiasis currently exists; the drug of choice for treating amebiasis is metronidazole, which may have severe side effects. Additionally, some clinical strains of *E. histolytica* are less sensitive to metronidazole, suggesting the emergence of metronidazole-resistant strains [2]. *E. histolytica* trophozoites proliferate in the intestinal lumen and phagocytose the resident gut flora with a preference for some species like *Lactobacillus ruminus* [3]. At first glance, the interaction between *E. histolytica* and the gut microbiota can be perceived as a simple interaction between a predator and its prey. Over the last few decades, this perception has begun to change and some recent reviews in the field have emphasized the complex interaction that occurs between the parasite and gut microbiota [4,5,6]. However, the role of this interaction in the resistance of the parasite to environmental factors present in the gut which are tightly associated with the generation of reactive oxygen species (ROS) and reactive nitrogen species (RNS) and on the pathogenesis of amebiasis has just emerged. In this review, we will focus on this last aspect of the Entamoeba-gut microbiota interaction.

## 2. Survey Methodology

Literature searching aimed at collecting any published data about Entamoeba parasites and the gut microbiota, resistance to oxidative stress (OS) and nitrosative stress (NS), virulence, crosstalk between *E. histolytica*, and gut microbiota. We searched literature relevant to the topic of the articles using PubMed and Google Scholar. Key words such as gut microbiota, amebiasis, resistance to oxidative stress, resistance to nitrosative stress, virulence, and probiotics were used to search. Then, screened articles were used as references for this review.

## 3. The Human Large Intestine and Its Associated Microbiota

The human intestine is inhabited by more than 100 trillion bacteria representing 500 to 1000 species [7]. These bacteria lives in a mutualistic association with their host and play a crucial role in producing vitamins and other metabolites that are essential to the host [8]. The gut microbiota is closely related to human health and once the intestinal flora is disturbed (gut microbiota dysbiosis), a series of intestinal diseases, such as inflammatory bowel disease, and colorectal cancer can develop [9].

The bacterial microbiome of the adult human gut is colonized by Firmicutes and Bacteroidetes that represent 90% of gut microbiota [10]. The establishment of gut microbiota is a dynamic process that begins even before birth to reach a stable level between 2 and 5 years of age [7]. Various factors influence the composition of gut microbiota following birth including the mode of delivery, the diet (breastmilk vs. formula), hygiene, and antibiotic treatment [11]. Between childhood and adulthood, food culture, which significantly differs between developed and developing countries, has a strong impact on the bacterial microbiome [12]. In adulthood, the gut microbiota community is relatively stable but it can change with age and diet. For example, older people present less Bacteroides, Bifidobacterium, and Enterobacteriaceae in their gut and more Clostridium species compared with younger adults [13]. Indeed, dietary fiber were found to increase faecal abundance of Bifidobacterium and Lactobacillus species [14].

## 4. Change Occurring in the Large Intestine Microbiota Following Infection with *E. histolytica*

Over the last few decades, it has become evident that *E. histolytica*’s pathogenicity is directly linked to the parasite’s interaction with the gut microbiota [4]. This interaction is very selective as only those bacteria with the appropriate recognition molecules are ingested by the parasite [15]. It has been reported that association with specific intestinal bacteria changes the *E. histolytica* cell surface architecture [16,17] and that phagocytosis of pathogenic bacteria boosts *E. histolytica* cytopathogenicity, increases the expression of Gal/GalNAc lectin on the cell surface, and boosts cysteine proteinase activity and resistance to oxidative stress (OS) when *E. histolytica* trophozoites are co-cultured with the enteropathogenic *E.coli* (EPEC) O55 [18] or *Shigella dysenteriae* [19]. Finally, bacteria-induced augmentation of *E. histolytica* virulence seems to occur only when the trophozoites phagocytose intact live cells [15]. The gut flora of patients suffering from amebiasis shows a significant decrease in the population of *Bacteroides*, *Clostridium coccoides*, *Clostridium leptum, Lactobacillus*, and Campylobacter and an increase in Bifidobacterium, while there is no change in Ruminococcus compared to healthy patients [20]. Interestingly, the fecal microbiota composition can be used as a predictive tool of Entamoeba colonization with an accuracy of 79% [21]. Some of the taxa, like Clostridiales Ruminococcaceae or *Prevotella copri*, which were central for the identification of patients infected with Entamoeba, have been associated with inflammatory bowel disease [22,23]. It is still not clear how a specific gut microbiota becomes associated with patients infected by Entamoeba. It is possible that the colonization of the gut by Entamoeba is predisposed by the gut microbiota of the host. Certain species of bacteria may also prevent the development of Entamoeba as it has been suggested for the commensal Clostridia, segmented filamentous bacteria [24]. Alternatively, *E. histolytica* feeds preferentially on certain species of bacteria [3] which may allow other species to proliferate.

## 5. Response of *E. histolytica* to OS

ROS play a key role in eliciting OS response in cells. They are capable of damaging essential biomolecules in the cell such as DNA, proteins, lipids, and they primarily inhibit cellular functions. Once formed, ROS leads to the oxidative damage of proteins thereby affecting their structure and functional properties [25,26]. In the large intestine, the invading *E. histolytica* trophozoites encounter OS. The sources of these stresses are fluctuations in oxygen tension in the intestinal lumen and the generation of ROS by cells of the immune system. Anti-amebic drugs like metronidazole and auranofin also induce oxidative damage of proteins by inhibiting thioredoxin reductase, a central enzyme in the protection of the parasite against OS [27]. Hydrogen peroxide (H_2_O_2_) is capable of damaging proteins by its interaction with thiol groups, which are present in the cysteine side chains as well as with metal cofactors. *E. histolytica* lacks antioxidant enzymes, such as catalase, glutathione reductase, and γ-glutamyl transpeptidase [28]. Thus, proteins such as the 29-kDa peroxiredoxin [29] and iron-containing peroxide dismutase [30] aid in OS resistance. It has been showed that *E. histolytica* strains sustain the exposure to OS better than avirulent strains, due to the presence of peroxiredoxin [31,32]. OS resistance contributes to the pathogenic potential of *E. histolytica* [33]. Additionally, OS leads to the oxidation of hundreds of proteins in the parasite including proteins involved in redox homeostasis, lipid metabolism, small molecule metabolism, carbohydrate derivative metabolism, and organonitrogen compound biosynthesis [34,35]. Oxidation of these proteins often lead to their inhibition as reported for glycolytic enzymes [36], virulence factors like the Galactose/*N*-acetylgalactosamine lectin which is essential for the binding of the parasite to host cells [35], and arginase, an enzyme that catalyzes the conversion of l-arginine to l-ornithine [35], a precursor of polyamine synthesis [37]. Polyamines and their biosynthetic enzymes are considered essential for growth and survival of unicellular parasites including Trypanosoma, Leishmania, and Plasmodium [37]. One of these polyamines, putrescine, has been linked to OS resistance and one of the proposed mechanism of OS resistance is based on its polycationic nature that enables it to couple with nucleic acids and membrane phospholipids. Putrescine is also free radical scavenger and an antioxidant [38]. The importance of putrescine and other polyamines in the resistance of *E. histolytica* to OS has been proposed [35] but direct experimental evidences to support this suggestion are still missing. OS induces a strong inhibition of protein synthesis in the parasite [35]. Although the mechanism for this inhibition is still not understood, it probably involves a higher eukaryotes in the phosphorylation of the initiation factor (eIF-2α) [39,40] and the oxidation of components of the parasite’s translational machinery, such as ribosomal proteins and elongation factors which leads to their inhibition [35,41]. At the transcriptomics level, OS triggers a complex response in the parasite which involves the modulation of a large number of genes which encode proteins with roles in translation, signaling/regulatory processes, metabolic/repair processes, energy metabolism, stress response, and transport [18,42]. The regulation of expression of genes which are responsive to OS mediated by H_2_O_2_ is controlled by a transcription factor EHI_108720 that binds to the AAACCTCAATGAAGA motif which is enriched in promoters of H_2_O_2_-responsive genes [43].

## 6. Response of *E. histolytica* to OS in Presence of Bacteria

### 6.1. Effect of Bacteria on E. histolytica Transcriptome

It was proposed more than 30 years ago that bacteria can compensate the lack of antioxidant enzyme in *E. histolytica* by complementing the parasite with such enzymes [15]. Excluding this work, the knowledge about the role of the gut microbiota on the resistance of the parasite to OS was scant. Unexpected interactions between the parasite and the bacteria that contribute to the resistance of the parasite to OS has been recently highlighted. Interaction of *E. histolytica* with *E. coli* O55 (ratio 1:1000) confers resistance of the parasite to OS [18]. At the transcriptomic level, *E. coli* O55 has almost no effect on gene expression in the parasite. However, when the parasite is exposed to *E. coli* O55 and to OS, the combination of these two stimuli triggers a strong transcriptomic response that involves almost 50% of the parasite’s coding gene [18]. This transcriptomic response is very different to the response of the parasite exposed to the OS alone. A general pattern of this combined response is the “normalization” of the level of expression of many genes that have been downregulated (including many ribosomal proteins) or upregulated (including oxidoreductases and several metabolic enzymes like glyceraldehyde-3-phosphate dehydrogenase and malate dehydrogenase) by OS. Downregulation of ribosomal proteins expression is a conserved mechanism to shut down unnecessary protein synthesis during stress [44]. In contrast, the upregulation of oxidoreductases and metabolic enzymes expression is a mechanism that compensate the inhibition of activity of these essential enzymes for the parasite following their oxidation [35]. The same “normalization” mechanism on gene expression in the parasite has been observed with two other bacteria, *Salmonella enterica* and *Enterococcus faecalis* but not with the probiotic *Lactobacillus acidophilus*. It is possible that the production of H_2_O_2_ by *L. acidophilus* [45] is detrimental to the parasite already exposed to OS. The effect that bacteria have on gene expression in the parasite exposed to OS goes beyond the “normalization” mechanism described above. Many leucine-rich repeat (LRR) proteins that were downregulated in the presence of OS were upregulated in the presence of bacteria and OS [18]. These LRR proteins which belong to the BspA family of proteins present structural homologies with Toll-like receptors (TLRs). TLRs are usually expressed on sentinel cells such as macrophages and dendritic cells and are involved in the recognition of structurally conserved molecules derived from microbes [46]. The possibility that the ancient protozoan *E. histolytica* displays key characteristics of the antibacterial response present in higher eukaryotes has been recently discussed [6,18]. However, the strong homology of sequence between these LRR proteins will make very challenging the testing of their functionality as TLRs with the genetic tools that are actually available to manipulate gene expression in *E. histolytica* [47]. The recent success to make the CRISPR/Cas9 system work in *E. histolytica* at an episomal level provides hope for the future study of these LRR proteins [48].

### 6.2. Effect of Chemical Molecules Originating from Bacteria

#### 6.2.1. Short-Chain Fatty Acids (SCFAs)

Gut microbial dysbiosis causes changes in SCFAs production leading, for example, to liver diseases [49] and neurodegenerative disorders [50]. The effect of chemical molecules originating from bacteria on the physiology of Entamoeba parasites has been pioneered by a study on SCFAs and their role in inhibiting encystation [51]. SCFAs are the main metabolites produced in the colon by bacterial fermentation of dietary fibers and resistant starch [52]. SCFAs inhibit OS in mammalian cells [53] and limit the genotoxic effect of H_2_O_2_ [54]. Based on this information, it will be very interesting to test in future the effect of different SCFAs like butyrate or propionate on the resistance of the parasite to OS.

#### 6.2.2. Oxaloacetate

Alpha-keto acids pyruvate, oxaloacetate, and alpha-ketoglutarate have a good H_2_O_2_-scavenging activity [55]. The role of oxaloacetate produced by the enteropathogenic *E. coli* O55 in protecting *E. histolytica* against OS has been recently demonstrated [34]. Malate dehydrogenase (MDH), which catalyzes the formation of oxaloacetate from malate, is essential for the protective effect to OS that *E. coli* O55 confers to *E. histolytica*. Two mechanisms by which oxaloacetate is delivered to the parasite are possible: (i) Intrabacterial oxaloacetate reach the parasite by phagocytosis of the bacteria and (ii) secreted *E. coli* MDH are forming oxaloacetate in the environment and this oxaloacetate acts like a shield by scavenging H_2_O_2_ before it affects the parasite’s viability. Oxaloacetate also has a role in promoting the virulence of the parasite, which confirmed previous observations about the correlation between virulence of the parasite and its resistance to OS [33]. In future, it will be interesting to test the protective effect of other alpha keto-acids produced by the microbiota on the resistance of the parasite against OS. Other antioxidant metabolites are produced by the gut microbiota like glutathione and folic acid [56]. *Entamoeba histolytica* lacks glutathione reductase activity, the ability to synthesize glutathione de novo and the ability to form trypanothione from taken up glutathione [57]. Therefore, the relevance of glutathione produced by the gut microbiota to the resistance of the parasite to OS is probably weak. In contrast, folic acid is one of the vitamins, which is currently added to the culture media of *E. histolytica* [58]. In view of the ability of folic acid to scavenge free radical [59], it will be interesting to test its ability to protect the parasite against OS.

#### 6.2.3. Queuine

**Figure 1:**
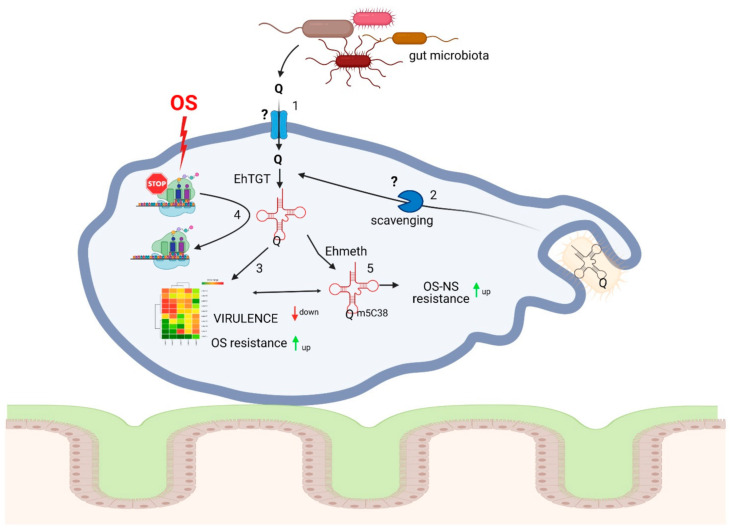
Queuine produced by the gut microbiota regulates *E. histolytica* resistance to oxidative stress (OS) and virulence. 1. Queuine which is produced in the human gut by the microbiota and released upon lysis of the bacteria can be salvaged by *E. histolytica*. The mechanism used to uptake queuine is unknown. 2. Queuosine-modified tRNAs present inside bacteria can enter inside the parasite following their phagocytosis. The parasite may rely on a dedicated enzymatic machinery to salvage Q. One possible candidate for this function is DUF2419 (EHI_098190), an *E. histolytica* protein with structural similarity with DNA glycosidases. Work is in progress to characterize the involvement of EhDUF2419 in the salvage of Q from bacteria. 3. Queuine regulates the transcriptome of the parasite by upregulating the expression of genes involved in the resistance to OS and by downregulating the expression of genes involved in virulence [66]. 4. Protein synthesis is impaired in the parasite exposed to OS [66]. In presence of queuine, OS does not block protein synthesis. This mechanism may help the parasite to stand OS. 5. Queuosine-modified tRNAs are hypermethylated by Ehmeth, the amebic homolog of mammalian Dnmt2 [66]. Hypermethylation of tRNA^Asp^_GUC_ has been correlated with the resistance of the parasite to OS and nitrosative stress (NS) [71,72]. It is possible that regulatory mechanisms described in 3 and 5 are linked [66]. This document has be created with BioRender.com (accessed on 10 March 2021).

Queuine and 7-(((4.5-cis-dihydroxy-2-cyclopenten-1-yl)-amino)-methyl)-7-deazaguanosine (queuosine—Q) are produced by bacteria. Q and its glycosylated derivatives occur in position 34 of the anticodon of tRNA^Asp^, tRNA^His^, tRNA^Asn^, and tRNA^Tyr^ of eubacteria and eukaryotes except for *Saccharomyces cerevisiae* [60,61]. Q is highly conserved and found in plants, fishes, insects, and mammals. While many bacteria can synthesize queuine (the nucleobase of Q) de novo, salvaging the prokaryotic Q precursors preQ_0_ and preQ_1_ has recently been reported [62]. Eukaryotes are not capable of Q synthesis and they rely on salvaging the queuine base as a Q precursor either by nutrition or by the intestinal bacterial flora [63,64,65]. The effects of queuine on the physiology of *E. histolytica* have been recently studied [66] and the main conclusions of this study are summarized in Figure 1. Queuine protects the parasite against OS and it antagonizes the negative effect that OS has on translation by inducing the expression of genes involved in the OS response like heat shock protein 70 (Hsp70), antioxidant enzymes such as alcohol dehydrogenases, and proteins involved in the repair of oxidative DNA damage like RecQ helicase. On the other hand, queuine impairs *E. histolytica* virulence by downregulating the expression of cysteine proteases and other genes associated with virulence [66]. This is the first example in Eukaryotes of an effect of queuine on the regulation of gene expression. In contrast to oxaloacetate and other alpha-keto acids that rely on their ability to scavenge H_2_O_2_ to protect *E. histolytica* against OS, queuine uses a much more complex mechanism that depends on tRNA-guanine transglycosylase (TGT) activity. TGT is the main enzyme responsible for the formation of Q in the anticodon loop position 34 of tRNA^Asp^, tRNA^His^, tRNA^Asn^, and tRNA^Tyr^. The enzyme exchanges G34 for the precursors. In contrast to eubacterial TGT enzymes, all of which are homodimers, eukaryotic TGT enzymes, such as human TGT, are heterodimers and consist of a Q tRNA-ribosyltransferase 1 (QTRT1) and a Q tRNA-ribosyltransferase domain-containing 1 (QTRTD1) [67,68]. *E. histolytica* TGT enzyme has been recently identified and forms a heterodimer composed of *Eh*QTRT1 and *Eh*QTRTD1. EhTGT is catalytically active and incorporates queuine into *E. histolytica* tRNAs. Two mechanisms can possibly explain why queuine protects the parasite against OS. The first mechanism relies on the reprograming of gene expression in the parasite exposed to queuine. Genes involved in the resistance to OS like heat shock protein 70 (Hsp 70), antioxidant enzymes like alcohol dehydrogenases 2, and DNA repairing enzymes like RecQ helicases have their expression upregulated in the presence of queuine [66]. Why queuine leads to a reprograming of these genes is still an open question. It can be the result of an increased transcription of these genes triggered by transcription factor(s) and/or by an accumulation of these mRNAs in the parasite cultivated in presence of queuine. Work is in progress to address this question. In the second mechanism that relies on studies performed in *S.pombe* and mammals, Dnmt2 activity is stimulated by prior queuosine incorporation at G34 of tRNA^Asp^_GUC_ [69,70]. Q-modified tRNA^Asp^_GUC_ is protected against endonuclease cleavage and it is therefore preferentially used by the cells for the translation of stress proteins. Data supporting the presence of this mechanism is *E. histolytica* which includes: (i) The exogenous supplementation of *E. histolytica* trophozoites with queuine leads to hypermethylation of C38 in tRNA^Asp^_GUC_ and (ii) hypermethylation of tRNA^Asp^_GUC_ catalyzed by the *E. histolytica* Dnmt2 homolog Ehmeth correlates with the resistance of the parasite to OS [71]. The two mechanisms may be connected as U (U-GUN) ending codons which are overrepresented in genes upregulated in the parasite exposed to queuine including possible transcription factors and proteins involved in OS resistance [66].

## 7. Response of *E. histolytica* to Nitrosative Stress (NS) and the Gut Microbiota

Following host invasion, the invading *E. histolytica* trophozoites are exposed to nanomolar concentrations of nitric oxide (NO) that is produced in intestinal epithelial cells by constitutive NO synthase [73] and as an intermediate in denitrification by the intestinal microbiota [74]. Although exposure to low NO concentrations is insufficient to kill the parasite [75], these low concentrations may strengthen its resistance to high NO concentrations. Amebiasis is characterized by acute inflammation of the intestine with the release of cytokines, such as tumor necrosis factor α, interleukin 8, interferon gamma, and interleukin β, and the generation of micromolar concentrations of ROS (discussed above) and reactive nitrogen species (RNS) from activated cells of the host’s immune system. NO in micromolar concentrations is cytotoxic for *E. histolytica*, and this cytotoxicity is implemented by *S*-nitrosylation of key metabolic enzymes and by fragmenting the endoplasmic reticulum (ER) [76,77]. NO also inhibits cysteine proteases [77], which are involved in differentiation, amino acid anabolism, inactivation of the host inflammatory response, lysosomal transport, and invasion of the host’s tissues [78]. NO can also regulate the activity and function of proteins by *S*-nitrosylation of their cysteine residues [79]. A high-throughput proteomic analysis of *S*-nitrosylated (SNO) proteins in NO-exposed *E. histolytica* using resin-assisted capture of SNO proteins [75], found that SNO proteins are involved in glycolysis, translation, protein transport, and virulence. *E. histolytica* can adapt to various stresses [80,81,82] including to progressive increases in the intestinal NO concentration [83], which may occur in patients with inflammation of the large intestine [73] or during the establishment of amebiasis [84].

Information about the role of the gut microbiota in protecting the host against NS is scanty. The role of acetate and butyrate, two SCFAs produced by the gut microbiota, to reduce NS in human islets and β cells after exposure to the apoptosis inducer and metabolic stressor streptozotocin [85] is one of the few examples available in the literature. In contrast, the ability of the gut microbiota to generate RNS is well discussed (for a recent review see [86]). The gut bacteria can convert nitrites into nitrosamines which have carcinogenic properties [87] and some food components present in meat and fish into trimethylamine. In the liver, trimethylamine is converted to its oxidized form (trimethylamine *N*-oxide) which have deleterious effects on cardiovascular and metabolic function [88].

Regarding *E. histolytica*, we did not found any protective effect of *E. coli* O55 on the resistance of the parasite to NS [18]. The lack of protection may be explained by the fact that *E. coli* O55 was not exposed to NS prior to its interaction with the parasite. *E. coli* possesses three major enzymes to overcome NS: the soluble flavohaemoglobin Hmp, the di-iron-center flavorubredoxin NorV with its NADH-dependent oxidoreductase NorW (NorVW) and the cytochrome c nitrite reductase NrfA. The expression of these enzymes is induced by the exposure of the bacteria to NS [89]. Consequently, it will be interesting to measure the effect of *E. coli* O55 on the resistance of *E. histolytica* to NS by using bacteria pre-exposed to NO. We have also addressed the role of queuine in protecting the parasite against NS. Queuine did protect the parasite against NS to some extend but the variability of the results among different experiments was very high (unpublished data).

## 8. *E. histolytica* Infection and Probiotics

Probiotics are live microorganisms that are intended to have health benefits when consumed or applied to the body [90]. It has been proposed that the use of probiotics, may present as complementary or as an alternative to the current treatment of amoebiasis. The possible effect of probiotics in preventing amebiasis has been recently reviewed [91]. A number of studies have been conducted to test the effectiveness of the probiotic at inhibiting adhesion of the protozoa to the intestinal mucosa surface [92,93]. More recently, it has been proposed that *Lactobacillus acidophilus* [18], *Lactobacillus casei* and *Enterococcus faecium* [94] are potent probiotics that can be used to fight amebiasis. How these probiotics work against the parasite is still not well understood. For *L. acidophilus*, it has been suggested that the ability of this bacteria to produce H_2_O_2_ [45] contributes to its amebicidal activity [18]. For *Weissella paramesenteroides* WpK, another lactic acid bacteria, amoebic lesions caused by *Entamoeba dispar* are reduced in presence of this bacteria. The authors proposed that *W. paramesenteroides* WpK4 works by strengthening the barrier function of the caecal mucosa [95].

## 9. Concluding Remarks

Beyond the predator-prey relationship that exists between the parasite and the gut microbiota evidences for a more complex interaction have emerged in the last decades. It is still not clear if the microbiota is paving the way for the development of amebiasis or if the disease is triggered by the dysbiosis caused by the parasite. It is probable that both scenarios are taking place. Small molecules originating from the bacteria like oxaloacetate, SCFA and queuine have proved to be important mediators between the bacteria and the parasite. These bacterial molecules which can control the different aspects of the physiology of the parasite may be exploited to manipulate the parasite and fight it. For example, the fact that queuine inhibits the virulence of *E. histolytica* may lead to new strategies for preventing and/or treating amebiasis by providing queuine to the host as a postbiotic (soluble factors secreted by live bacteria, or released after bacterial lysis that can be used to improve host health [96]) or via probiotics. Such strategy has been proposed for example with the gut bacteria *Gordonibacter pamelaeae* that produces the anticarcinogen urolithin [97]. SCFA, oxaloacetate and queuine represent probably the top of the iceberg of the molecules used between the microbiota and the parasite to communicate. It is essential to perform a systematic screen for such molecules in the future. Many challenges in studying the microbiome in the context of human diseases exists including the choice of appropriate experimental systems [98]. These challenges exist also in the study of the role of the microbiota in amebiasis. It is essential in the future to develop a simple model to study the interaction of the microbiota with the parasite in the gut. One such model that we are currently investigating is a three-dimensional intestinal model that replicates the general characteristics of the human colon. This model has been recently used to investigate the early stage of invasion of the gut by *E. histolytica* trophozoites [99]. Finally, what can be learned from the interaction taking place between *E. histolytica* and the microbiota is certainly relevant to other parasitic protozoa and helminths which are also in a tight relationship with the host’s intestinal microbiota. For example, the antioxidant properties of oxaloacetate which is produced by the gut microbiota is also valid for the protection of *C*. *elegans* by oxaloacetate against H2O2-induced oxidative stress [34].
